# Supplementary material for: Iterative improvement in the automatic modular design of robot swarms
Source: PeerJ Comput Sci. 2020 Dec 7;6:e322. doi: 10.7717/peerj-cs.322 (PMC7924708; doi:10.7717/peerj-cs.322)
Supplement: Supplemental Information 3 [file peerj-cs-06-322-s003.zip › argos3/doc/api/standalone/a00507_source.html]

ARGoS: plugins/robots/generic/control\_interface/ci\_positioning\_sensor.h Source File


- Main Page
- Related Pages
- Namespaces
- Classes
- Files

- File List
- File Members

# plugins/robots/generic/control\_interface/ci\_positioning\_sensor.h

Go to the documentation of this file.

```
00001 
00006 #ifndef CCI_POSITIONING_SENSOR_H
00007 #define CCI_POSITIONING_SENSOR_H
00008 
00009 namespace argos {
00010    class CCI_PositioningSensor;
00011 }
00012 
00013 #include <argos3/core/control_interface/ci_sensor.h>
00014 #include <argos3/core/utility/math/vector3.h>
00015 #include <argos3/core/utility/math/quaternion.h>
00016 
00017 namespace argos {
00018 
00019    class CCI_PositioningSensor : public CCI_Sensor {
00020 
00021    public:
00022 
00023       struct SReading {
00024          CVector3 Position;
00025          CQuaternion Orientation;
00026       };
00027 
00028    public:
00029 
00030       virtual ~CCI_PositioningSensor() {}
00031 
00032       const SReading& GetReading() const;
00033 
00034 #ifdef ARGOS_WITH_LUA
00035       virtual void CreateLuaState(lua_State* pt_lua_state);
00036 
00037       virtual void ReadingsToLuaState(lua_State* pt_lua_state);
00038 #endif
00039 
00040    protected:
00041 
00042       SReading m_sReading;
00043       CRadians m_cAngle;
00044       CVector3 m_cAxis;
00045 
00046    };
00047 
00048 }
00049 
00050 #endif
```

---

Generated on 10 Jul 2018 for ARGoS by 
 1.6.1
